# Comparative Assessment of Neutrophil Gelatinase-Associated Lipocalin (NGAL) and Cystatin C as Early Biomarkers for Early Detection of Renal Failure in Patients with Hypertension

**DOI:** 10.6091/ibj.1380.2015

**Published:** 2015-04

**Authors:** Fatemeh Gharishvandi, Faranak Kazerouni, Esmat Ghanei, Ali Rahimipour, Malihe Nasiri

**Affiliations:** 1 *Dept. of Laboratory Medicine, Faculty of Paramedical Sciences, Shahid Beheshti University of Medical Sciences, Tehran, Iran;*; 2 *Dept. of Internal Medicine, Shohada Tajrish Hospital, Shahid Beheshti University of Medical Sciences, Tehran, Iran;*; 3 *Dept. of Biostatics, Faculty of Paramedical Sciences, Tarbiat Modares University, Tehran, Iran*

**Keywords:** Neutrophil gelatinase-associated lipocalin (NGAL), Cystatin C, Creatinine, Hypertension

## Abstract

**Background:**

Hypertension is one the most common causes of chronic kidney disease (CKD). One of the major concerns in hypertensive patients is early detection of renal disorders. In the past, serum creatinine (Scr) concentration was used as a marker of kidney function, but it proffers a late reflection of reduced glomerular filtration rate. Cystatin C and neutrophil gelatinase-associated lipocalin (NGAL) have been recently proven to be useful for quantification of CKD. Therefore, we compared the diagnostic value of NGAL with cystatin C and creatinine to evaluate kidney function in hypertensive patients.

**Methods:**

In this study, 42 hypertensive patients and 30 healthy volunteers were recruited. Serum cystatin C (Scys C) and plasma NGAL were measured using ELISA method. Creatinine, urea, hemoglobin, fibrinogen, and C-reactive protein were measured according to the routine methods. Estimated glomerular filtration rate (eGFR) was considered as the gold standard method (cut-off value of < 78 ml/min/1.73 m^2^.

**Results:**

In the patient group, plasma NGAL, cystatin C, and creatinine were all significantly correlated with eGFR, and plasma NGAL correlated best with eGFR. Receiver-operating characteristics analysis indicated that plasma NGAL was a better indicator than creatinine and cystatin C for predicting a GFR < 78 ml/min/1.73 m^2^. The sensitivity and specificity for NGAL were 96% and 100%, for cystatin C were 92% and 60% and for creatinine were 76% and 47%, respectively.

**Conclusion:**

Plasma NGAL demonstrated a higher diagnostic value to detect kidney impairment in the early stages of CKD as compared to Scys C and Scr in hypertensive patients.

## INTRODUCTION

Chronic kidney disease (CKD) is defined as either kidney damage or decreased kidney function (decreased eGFR [estimated glomerular filtration rate]) for at least three months [1]. Hypertension is one of the risk factors for CKD; therefore, an early detection of renal impairment is one of the major concerns in these patients. Serum creatinine (Scr) is an inadequate marker for determination of kidney function, since its concentration does not change significantly until creatinine clearance is less than 70 ml.min.1.73 m^2^. Besides, Scr level is affected by factors, such as body mass, age, race, gender, inflammation as well as certain drugs such as cimetidin [2, 3].Studies have suggested that serum cystatin C (Scys C) is a more accurate predictor of kidney function in comparison to Scr [4]. Cystatin C is an endogenous marker that is freely filtered in the glomeruli. Similar to other low molecular mass proteins, cystatin C is almost completely reabsorbed by tubular epithelial cells and subsequently catabolized so that it does not return to the blood flow. In addition, it is not influenced by inflammation, muscle mass and does not face the same problems with analytical interferences as creatinine [5, 6]. Another potential biomarker, neutrophil gelatinase-associated lipocalin (NGAL), is a member of lipocalin family and a ubiquitous 25-kD protein [7]. According to some new hypotheses, NGAL release from renal tubule occurs soon after the damage notably preceding the rise in Scr [8-12]. In addition to some research works that described NGAL along with some already known factors as a independent risk marker for progression of CKD disease [13, 14], Mitsnefes *et al.* [15] suggested that NGAL could be used as a biomarker of kidney disease and severity. In the present study, we investigated the potential application of plasma NGAL (pNGAL) as an early biomarker of kidney impairment in hypertensive patients and then compared its diagnostic power with Scys and Scr.

## MATERIALS AND METHODS

In this cross-sectional study, 42 patients (10 men and 32 women) with high blood pressure (systolic and diastolic blood pressure ≥ 140 and ≥ 90 mmHg, respectively [16]) were recruited. Volunteers were selected from patients who referred to the Shohada Tajrish Hospital (Tehran, Iran). Mean age of the hypertensive patients was 54.33 ± 8.9 years, and their high blood pressure was confirmed by a doctor at least in two separate occasions.

For reduction of potential confounding factors, patients with chronic diseases, such as diabetes, liver and cardiovascular diseases, and elevated Scr and urea were excluded from the study. All patients were informed about the aim and procedure of the study and gave their informed consent. Healthy volunteers (n = 30) with the mean age of 54.73 ± 6.85 years were selected as the control group. Blood samples were collected in the morning before any food intake. Biochemical parameters including urea, Scr, hemoglobin, fibrinogen and C-reactive protein (CRP) were measured according to the standard methods in the routine clinical laboratory. eGFR (estimated creatinine clearance) were calculated using Cockcroft-Gault formula [17]:

eGFR (ml/min)=(140– Age)× mass (kg)×(0.85 if female)72× Scr(mg/dl)

All clearances were expressed as ml/min/1.73 m^2^ after correction for body surface area according to the DuBois-DuBois formula [18]: 

Body surface area (m^2^) = 0.007184 × height (cm)^0.725^ × weight (kg)^0.425^

 For pNGAL measurement, blood was placed into chilled vacutainer tubes containing potassium ethylenediaminetetracetate, and the plasma was promptly separated in a refrigerated centrifuge (1,820 ×g, at 4^○^C, 5 min) [19, 20]. The samples were stored at -20^○^C until assay, and pNGAL was evaluated using commercially available ELISA kit (Biovender, Norway). The intra- and inter-assay variances for pNGAL were 7.7% and 9.8% respectively. All measurements were made in a triplicate and in a blinded manner. pNGAL levels were expressed as nanograms per milliliter [21]. Scys was estimated using ELISA method (Biovender, Norway), and all tests were performed according to the manufacturer's instructions.

***Statistical analysis. ***Statistical analysis was performed using SPSS software (version18). Data were expressed as mean ± SD or percentage.* t*-test was used for comparison of the means between the two groups. Correlation between eGFR and other variables were assessed by Pearson's coefficient. The maximum efficiency, sensitivity, specificity, and positive- and negative-predictive values were also calculated. Receiver-operating characteristics analysis was used to calculate the area under the curve for pNGAL, Scys, and Scr to find the best NGAL, Scys, and Scr cut-off values for identifying the patients at risk of CKD. The accuracy of detection of pNGAL, Scys, and Scr was calculated using chi-square test. All results were considered significant if *P* value was < 0.05.

## RESULTS

 This study was performed on 42 high blood pressure patients (10 men and 32 women) with the mean age of 54.33 ± 8.89 years and 30 healthy individuals (14 men and 16 women) with the mean age of 54.7 ± 6.8 years. As shown in Table 1, the levels of pNGAL, Scys, Scr, and eGFR were significantly higher in the patients compared to the control group (*P* < 0.05).

**Fig. 1:**
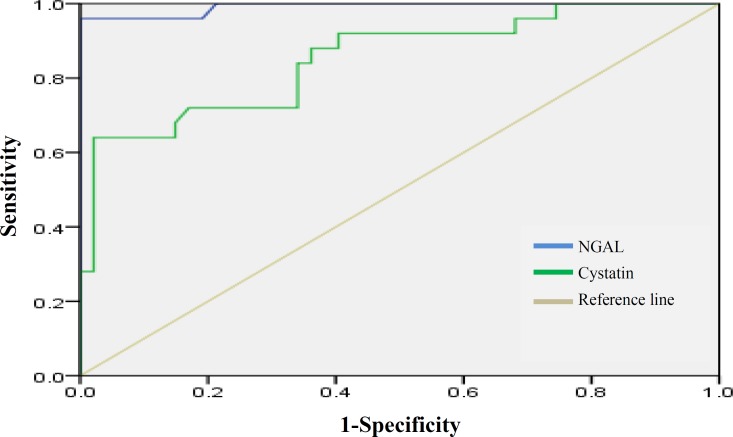
Receiver-operating characteristics analysis showing plasma neutrophil gelatinase-associated lipocalin (NGAL), serum cystatin C (Scys) and serum creatinine (Scr) as indicators of GFR < 78 ml/min/1.73 m2.

 In this study, using the Pearson's correlation coefficient, eGFR correlation with various parameters, including urea, fibrinogen, CRP, hemoglobin, pNGAL and Scys was assessed. From the above parameters, eGFR showed a significant inverse correlation with pNGAL (R = -0.593, *P *< 0.001), Scys (R _=_ -0.453, *P* < 0.001), and Scr (R _=_ -0.251, *P *_=_ 0.033 ), but no correlation was observed between eGFR and urea (R _= _0.01, *P *_= _0.8), eGFR and fibrinogen (R _= -_0.105, *P *_=_ 0.3), eGFR and hemoglobin (R _= _0.129, *P*_ = _0.2), and GFR and CRP (R _= -_0.068, *P *_= _0.5). Receiver-operating characteristics analysis (Fig. 1) indicated that pNGAL was a better indicator than Scr and Scys for predicting a GFR < 78 ml/min/1.73 m^2^. The sensitivity and specificity were 96% and 100% for pNGAL (≥32.2 ng/ml) compared with 76% and 47% for sCr (≥0.97 mg/dl) and 92% and 60% for Scys, respectively (≥980 ng/ml). The best cut-off values of pNGAL, Scys, and Scr for detection of eGFR < 78 were 32.2 ng/ml, 980 ng/ml, and 0.97 mg/dl, respectively. 

**Table 1:** Comparison of the mean pNGAL, Scys, Scr, eGFR, systolic blood pressure, diastolic blood pressure, hemoglobin, fibrinogen, CRP, and urea in the patient and control group

Variables	Control group	Patient	*P* value
	(n = 30)	(n = 42)	
**pNGAL (ng/ml)**	**14.59 ± 3.71**	**124.54 ± 118.67**	**< 0.001**
**Scys (ng/ml)**	**829.27 ± 295.65**	** 1120.9 ± 229.10**	**< 0.05**
**Scr (mg/dl)**	**0.97 ± 0.136**	** 1.058 ± 0.18**	**0.036**
**eGFR (ml/min/1.73m²)**	**90.74 ± 10.38**	**77.73 ± 20.19**	**0.001**
**Systolic blood pressure**	**110.50 ± 10.5** **0**	** 160.5 ± 18 mmHg**	**< 0.01**
**Diastolic blood pressure**	**60.50 ± 11.00**	** 90.7 ± 9.7 mmHg**	**< 0.01**
**Hemoglobin** ** (g/dl)**	**13.41 ± 0.98**	** 13.19 ± 0.96**	**0.33**
**Fibrinogen (mg/dl)**	**220.00 ± 66** **.00**	** 212 ± 6.00**	**0.59**
**CRP (mg/dl)**	**0.44 ± 0.15**	** 0.46 ± 0.2** **0**	**0.56**
**Urea (mg/dl)**	**30.90 ± 7.26**	** 24.38 ± 8.16**	**0.01**

pNGAL, plasma neutrophil gelatinase-associated lipocalin; Scys, serum cystatin; Scr, serum creatinine; eGFR, estimated glomerular filtration rate; CRP, C-reactive protein

 As shown in Figure 1, the percent under the receiver operating characteristic curve for pNGAL was 99%, which is greater than Scr and Scys.

Distribution of the patients based on pNGAL levels and eGFR is given in Table 2, indicating that positive- and negative-predictive values of pNGAL are 96% and 94%, respectively. In addition, 2.3% false-positive results and 2.3% false-negative results were found when pNGAL was used for detecting impaired renal function in these patients.

 Distribution of the patients based on Scys levels and eGFR is given in Table 3, which displays that positive- and negative-predictive values of Scys are 79% and 84.6%, respectively. Moreover, 14.2% false-positive results and 4.6% false-negative results were found when Scys was used for detecting impaired renal function in these patients.

Distribution of the patients based on Scr levels and eGFR is given in Table 4, showing that positive- and negative-predictive values of Scr are 70.4% and 60%, respectively. Also, 19% false-positive results and 14.2% false-negative results were found when Scr was used for detecting impaired renal function in these patients. Distribution of the subjects in terms of the accuracy of detection of pNGAL, Scys, and Scr to determine eGFR < 78 is presented in Table 5. The Table shows incorrect diagnosis in 14 cases using Scr (33.2%), 2 cases using pNGAL (4.6%), and 8 cases using Scys (8.8%).

**Table 2:** Distribution of the patients according to diagnosis with pNGAL and eGFR

eGFR (ml/min/1.73m²)	(%) Count(-)	(%) Count( +)	Total
pNGAL (ng/ml )			
**NGAL < 32.2**	**16**	**1**	**17**
** (-)**			
**eGFR ≥ 78**	**94.1%**	**4.0%**	**40.5%**
**NGAL ≥ 32.2**	**1**	**24**	**25**
** (+)**			
**eGFR < 78**	**5.9%**	**96.0%**	**59.5%**
**Total**	**17** **100%**	**25** **100%**	**42** **100%**

Sensitivity = 24/25 = 96%; specificity = 100%; positive predictive value = 24/25 = 96%; negative predictive value = 16/17 = 94%, estimated glomerular filtration rate; pNGAL, plasma neutrophil gelatinase-associated lipocalin;

## DISCUSSION

 Studies have shown that there is an increased risk for CKD among individuals with high blood pressure [22, 23] An early marker of kidney damage would promote earlier intervention in order to arrest the progression to end-stage renal disease. Fortunately, the application of advance technologies has identified candidates that are emerging as early biomarkers of CKD. One such promising biomarker is NGAL [24]. In the current study, pNGAL levels were significantly higher in the hypertensive patients affected by non-advanced CKD with stable renal function compared to the control group, similar to what has been described previously [19]. The results from this study showed a significant inverse correlation with pNGAL, Scys, and Scr. This inverse correlation was more significant for pNGAL as compared to Scys and Scr. Consistent with our finding, Bolignano *et al*. [19] found a significant inverse correlation between eGFR and pNGAL; however, unlike our study, they found a significant correlation between eGFR and CRP as well as fibrinogen and hemoglobin, probably because patients in Bolignano's study [19] were in more advance stages of kidney damage. Another study [15] on subjects with CKD stages 2-4 also demonstrated that pNGAL concentr-ations were inversely associated with GFR and as kidney function declined to less than 30 ml/min, NGAL was a better biomarker of kidney failure compared to cystatin C. In contrast to our result, in Szewczyk *et al. *[25] study no significant correlation was found between eGFR and NGAL. It seems that in their research, confounding factors such as diabetes and other chronic disease which could contribute to increased NGAL levels were not excluded [6]. 

**Table 3:** Distribution of the patients according to diagnosis with Scys and eGFR

** eGFR ** **(ml/min/1.73m²)**	**(%) Count (-)**	**(%) Count (+)**	**Total**
**Scys ( ng/ml)**			
(-) Scys < 980 eGFR ≥ 78	11 64.7%	2 8.0%	13 31.0%
(+) Scys ≥ 980 eGFR < 78	6 35.3%	23 92.0%	29 69.0%
Total	17 100%	25 100%	42 100%

Sensitivity = 23/25 = 92%; specificity = 60%; positive predictive value = 23/29 = 79.3%; negative predictive value = 11/13 = 84.6%, eGFR, estimated glomerular filtration rate; Scys, serum cystatin;

**Table 4:** Distribution of the patients according to diagnosis with Scr and eGFR

eGFR (ml/min/1.73m²)	(%) Count (-)	(%) Count (+)	Total
Scr (mg/dl)			
(-) Scr < 0.97 eGFR ≥ 78	9 52.9%	6 24.0%	15 35.7%
(+) Scr ≥ 0.97 eGFR < 78	8 47.1%	19 76.0%	27 64.3%
Total	17 100%	25 100%	42 100%

Sensitivity = 19/25 = 76%; specificity = 47%; positive predictive value = 19/27 = 70.4%; negative predictive value = 9/15 = 60%, eGFR, estimated glomerular filtration rate; Scr, serum creatinine

Receiver-operating characteristics curves indicated that the diagnostic potential of pNGAL to detect patients with a GFR less than 78 ml/min/1.73 m^2^ was superior to Scr and Scys. Based on the results from this study, the best cut-off value for pNGAL to predict early stages of CKD was found to be 32.2 ng/ml with the sensitivity of 96% and specificity of 100%. pNGAL cut-off value in the current investigation is much lower than a previous investigation [26], most likely because patients in our study were at their early stages of CKD.

In accordance to a previous study, our results indicate the diagnostic superiority of Scys to Scr for predicting eGFR < 78 ml/min/1 m^2 ^[27]. Furthermore, based on the given sensitivity and specificity, the percentage of false-negative and false-positive values of pNGAL were lower than Scys and Scr, suggesting the higher diagnostic power of pNGAL compared to Scys and Scr. Our results suggest that pNGAL represents an early biomarker of CKD. This increase in pNGAL production of NGAL by injured cells rather than decrease in renal protein clearance capacity of the cells [28]. It is suggested that serial rather than isolated single measurement of NGAL will provide the most useful data in patients affected by risk factors of CKD [29]. In conclusion, our results suggests pNGAL to be a potential early marker for detection of impaired kidney function with higher diagnostic power compared to Scr and Scys in hypertensive patients. 

**Table 5:** Accuracy of diagnosis of pNGAL, Scys, and Scr to determine eGFR < 78

**Biomarker **	**Yes**	**No**	**Total**
pNGAL	2 (4.6)	40 (95.4)	42 (100)
Scys	8 (18.8)	34 (81.2)	42 (100)
Scr	14 (33.2)	28 (66.8)	42 (100)

pNGAL, plasma neutrophil gelatinase-associated lipocalin; Scys, serum cystatin; Scr, serum creatinine; eGFR, estimated glomerular filtration rate
